# The Role of Autologous Stem Cell Transplantation in the Treatment of Diffuse Large B-Cell Lymphoma

**DOI:** 10.1155/2012/195484

**Published:** 2012-01-18

**Authors:** Marco Gunnellini, Rita Emili, Stefano Coaccioli, Anna Marina Liberati

**Affiliations:** ^1^Department of Transplant Oncohematology, Perugia University, S. Maria, Terni, Italy; ^2^Faculty of Medicine, Perugia University, S. Maria, Terni, Italy

## Abstract

Diffuse large B-cell non-Hodgkin's lymphoma (DLBCL) accounting for approximately 30% of new lymphoma diagnoses in adult patients. Complete remissions (CRs) can be achieved in 45% to 55% of patients and cure in approximately 30–35% with anthracycline-containing combination chemotherapy. The ageadjusted IPI (aaIPI) has been widely employed, particularly to “tailor” more intensive therapy such as high-dose therapy (HDT) with autologous hemopoietic stem cell rescue (ASCT). IPI, however, has failed to reliably predict response to specific therapies. A subgroup of young patients with poor prognosis exists. To clarify the role of HDT/ASCT combined with rituximab in the front line therapy a longer follow-up and randomized studies are needed. The benefit of HDT/ASCT for refractory or relapsed DLBCL is restricted to patients with immunochemosensitive disease. Currently, clinical and biological research is focused to improve the curability of this setting of patients, mainly young.

## 1. Introduction

Diffuse large B-cell non-Hodgkin's lymphoma (DLBCL) is the commonest histological subtype of non-Hodgkin's lymphomas (NHL) accounting for approximately 30% of new lymphoma diagnoses in adult patients. Because their incidence increases in old age, this epidemiological pattern might explain, at least in part, the rapid rise in the number of new diagnoses observed over the last decades of the 20th century [1, 2] in which an increase of median age of population has also been registered.

Complete remissions (CRs) can be achieved in 45% to 55% of patients and cure in approximately 30–35% with anthracycline-containing combination chemotherapy [3].

The International Prognostic Index (IPI) proposed in the 1993 [4] has been used in the risk stratification for patients with DLBCL for more than a decade. The age-adjusted IPI (aaIPI) has also been widely employed, particularly to “tailor” more intensive therapy such as high-dose therapy (HDT) with autologous hemopoietic stem cell rescue (ASCT). IPI, however, has failed to reliably predict response to specific therapies. This, in part, reflects the inherent biological heterogeneity of DLBCL and highlights the need for more precise, patient-specific, and biologically based risk factors. Despite these criticisms, the IPI has proved valuable for stratification of patients in clinical trials and remains the prognostic system more widely employed in clinical research and daily practice.

The development of rituximab, a chimeric anti-CD20 monoclonal antibody, has represented a revolutionary advance in the therapy of hematologic malignancies [5]. The addition of rituximab to cyclophosphamide, doxorubicin, vincristine, and prednisone (CHOP) combination has produced significant survival benefits in elderly patients with untreated DLBCL compared to CHOP alone [6, 7]. Similarly, the same immunochemotherapy regimen has determined an improved outcome in young low-risk DLBCL patients [8], as defined by aaIPI. Thus, first line chemotherapy with CHOP or CHOP-like regimens in combination with rituximab has become standard care for CD20+ DLBCL patients.

Despite the striking advances in the outcome of DLBCL patients, a subgroup of young patients with poor prognosis still exists [9, 10]. Currently, clinical and biological research is focused to improve the curability of this setting of patients, mainly young.

## 2. HDT with ASCT in Front-Line Treatment of DLBCL

In the prerituximab era, HDT/ASCT has proven effective as salvage treatment in patients with chemosensitive relapsed aggressive NHL [11]. These results suggested the possibility of improving the outcome of aggressive NHL patients by including HDT/ASCT in the first-line therapy. After some phase I/II trials supporting the use of this strategy, HDT/ASCT appeared a promising option for frontline treatment of young patients. However, the results of prospective randomized trials [12–25] have generated conflicting results and several problems have hampered the comparison of data (Tables 1 and 2).

Firstly, trials had different remission status requirements for HDT/ASCT [12–25]. In particular, only patients in PR or CR, after induction therapy (Table 1), were randomized to receive HDT/ASCT or conventional therapy [12–16]. Secondly, in other trials, patients were randomized at diagnosis (Table 2), and HDT/ASCT was employed as part of initial treatment after shortened [19–21, 25] or full course of induction therapies [17, 18, 22–24]. Furthermore, high-dose sequential (HDS) therapy, a type of induction treatment based on a different “philosophy” from the rationale underlying the conventional one, was administered up-front followed by HDT/ASCT in three studies [17, 23, 24]. HDS therapy consists in the administration of several non-cross-resistant drugs, each given at the maximal tolerated dose mainly as single agent within the shortest possible interval. The purpose of this regimen was to prevent the emergence of drug-resistant tumor clones. Thirdly, a great variety of therapeutic regimens, both among conventional or high-dose treatments, were employed. In fact, conventional CHOP regimen or CHOP-like combinations were employed in 3 and 6 trials, respectively, while, in the other studies, MACOP-B or VACOP-B were used [17, 18, 21, 22]. Although the combination of carmustine, etoposide, cytarabine, and melphalan (BEAM) was the most frequently employed conditioning regimen [15, 16, 18–20, 22, 25], other myeloablative treatments [12–14, 17, 21, 23, 24] were also used in several trials. Fourthly, because several of these studies were designed before the introduction in the clinical practice of both the IPI prognostic score [4] and the REAL-WHO histological classification [26], trials included varying proportions of patients with different risk categories and different histological subtypes, not all of which were DLBCL. Despite the poor comparability of these trials, a statistically significant prolongation of PFS or EFS was documented in four trials [15, 19, 22, 25], but none demonstrated a significant improvement of OS associated with HDT/ASCT with the exception of a retrospective subgroup analysis [14]. In summary, in the prerituximab era, HDT/ASCT, employed in front-line therapy, failed to improve the outcome of aggressive NHL patients.

In the rituximab era, HDT/ASCT for intermediate-high (I-H) or high-risk (H) aaIPI patients is still a matter of debate. However, the combination of rituximab with an intensified treatment strategy has resulted in encouraging results in phase II studies (Table 3). Tarella et al. [27] used rituximab in combination with modified HDS chemotherapy delivered with multiple ASCT followed by a consolidation phase consisting of mitoxantrone (Mito) and melphalan (L-PAM) with ASCT. In this study, 93 of the 112 patients enrolled completed the planned therapy. At conclusion of treatment, the CR rate was 80%. At a median followup of 48 months, the estimated 4-year OS projection was 76% (CI: 68–85%), and at median followup of 46 months, the 4-year EFS projection was 73% (CI: 64–81%). Vitolo et al. [28] employed 4 cycles of dose-dense (110 mg/mq epirubicin, 1200 mg/mq ciclofosfamide, 1.4 mg/mq vincristina, and 40 mg/mq prednisone orally days 1 to 5 given every two weeks) CEOP regimen as induction phase, followed by the 2 cycles of mitoxantrone, cytarabine, and dexamethasone (MAD) as intensification phase. The third phase of study design consisted of BEAM with ASCT. A total of six rituximab doses were given, 4 and 2 during induction and intensification phases, respectively. Seventy six of the 94 patients completed treatment and underwent HDT/ASCT. The CR rate was 82% (CI: 73–88%). With a median followup for censored patients of 49 months, the 4-year FFS rate was 73% (CI: 63, 5–82, 5%) and the 4-year OS rate was 80% (CI: 71, 6–88,4%). Dilhuydy et al. [29] reported an overall response (OR) rate of 67%. With a median followup of 66 months, the estimated rates (±SD) of 5-year OS and EFS rates were 74%  ±  4% and 55%  ± 5%, respectively. Fitoussi et al. [30] treated 208 patients with rituximab combined with cyclophosphamide, vindesine, bleomycin, and prednisolone (ACVBP) for 4 cycles. This induction therapy was followed by BEAM with ASCT in 155 responding patients (CR or PR). A total of 32 patients did not receive HDT/ASCT. Twenty five were withdrawn during induction therapy, 6 because of insufficient response before consolidation and one because of sudden death. With a median followup of 45 months, the 4-year PFS and OS were estimated at 76% (CI: 69–81%) and 78% (72–83%).

In both the Vitolo and the Fitoussi studies, the results achieved with the immunochemotherapy strategy were compared with those obtained in their historical groups of patients treated with similar sequence of chemotherapy program, but not including rituximab. Despite the limitations intrinsic to retrospective analyses, these comparisons showed a clear therapeutic advantage of immunotherapy over chemotherapy in both the two major end points PFS and OS.

Recently two randomized studies conducted by the SWOG [31] and FIL [32] have tested the role of HDT/ASCT in the front line therapy of unfavorable (I-H/H) patients with aggressive NHL. In particular, in the SWOG study, patients responsive to the CHOP or R-CHOP induction therapy were randomized to receive one more cycle of R-CHOP followed by TBI or BCNU-based regimens and ASCT or three additional cycles of R-CHOP [31]. In this trial, the 2 yr PFS was 69% and 56% in the experimental arm compared to the standard one (95% CI: 1.18–2.51) *P* = 0.05, while no significant difference was documented in the 2-year OS. The authors conclude that HDT/ASCT improves PFS for responders, including those induced with R-CHOP, with a stronger outcome seen for those with H IPI grade. The FIL study, a multicenter randomized trial with a 2 × 2 factorial design, compared two rituximab dose-dense treatments (R-CHOP14 versus R-megaCHOP14), followed or not by BEAM with ASCT [32]. With a median followup of 23 months, 2-year PFS was 65% (CI: 59–70%), for the entire group of enrolled patients and 59% (CI: 51–57%) versus 72% (CI: 64–78%) for no HDT/ASCT versus HDT/ASCT respectively. So far, the advantage in PFS does not translate in OS benefit. However, a longer followup will clarify the role of HDT/ASCT as first-line treatment of aaIPI 2-3 DLBCL patients. These and other randomized studies will define whether HDT/ASCT combined with rituximab in the front line therapy is associated with increased cure rate of unfavorable DLBCL patients.

## 3. HDT with ASCT as Salvage Therapy

In the prerituximab era, the Parma trial established HDT/ASCT as standard therapy in relapsing aggressive NHL patients responding to salvage therapy [11].

 The parameters affecting the results of HDT/ASCT are identified in responsive disease to conventional dose salvage therapy before myeloablative treatment, relapse defined as a time less than twelve months from diagnosis to recurrence (early), and the presence of prognostic factors at relapse, as defined by IPI or secondary aaIPI (saaIPI) [33–35].

At present, the emphasis of recent clinical research in HDT/ASCT is focused on three therapeutic aspects. The first consists in the evaluation of the potential benefit of adding rituximab to salvage therapy, followed by HDT/ASCT, in relapsed rituximab naïve patients. Overall, the available data in this setting of patients, although not completely concordant, are in favour of the use of immunochemotherapy (Table 4). In first three studies reported in Table 4, no patient [36] or only a minority of cases [37, 38] had received rituximab before enrollment, while, in the fourth, 25% of patients were treated with rituximab in the first line therapy or during salvage treatment or both at diagnosis and after relapse [39]. In the Kewalramani et al. study, the PFS rates of patients who underwent HDT/ASCT after ICE in combination with rituximab (RICE) were marginally improved compared to those observed in the historical control patients who received salvage therapy alone [36]. The difference was not statistically significant, but the study was not powered to detect minor improvements in survival rates. However, in this study, the addition of rituximab to ICE doubled the percentage of CRs. Sieniawski et al., in their study reported improved OR rate, freedom from second failure (FF2F), and OS in the patient group treated with DHAP plus rituximab, compared to the historical control group treated with the same chemotherapy [37]. In both groups, patients in CR or PR after salvage therapy received HDS therapy followed by BEAM with ASCT. Improved FFS and PFS were documented also by Vellenga et al. [38] in relapsed patients when rituximab was added to DHAP-VIM-DHAP reinduction therapy. The modest impact of rituximab on OS was amplified when the analysis was repeated adjusting for prognostic factors such as time elapse since upfront treatment, saaIPI score, age, and WHO PS. Furthermore, the addition of rituximab increased the group of responders on reinduction therapy from 54% to 74% and therefore the number of patients who might benefit from HDT/ASCT. In this study, as well, only patients in CR or PR after salvage therapy were eligible for HDT/ASCT. In the Mounier et al. study [39], after HDT/ASCT, the 5-year OS was 63% (95 CI, 58–67%), and the 5-year DFS was 48% (95 CI, 43–53%) for the entire population. Statistical analysis indicated a significant increase in DFS after ASCT compared with duration of CR I (median, 51 months versus 11 months; *P* < .001). This difference remained highly significant in patients with previous exposure to rituximab (median, 10 months versus not reached; *P* < 0.01). The second aspect regards the role of rituximab in salvage treatment of patients previously treated with immunochemotherapy (Table 5). In fact, at present, almost all patients with aggressive B-cell NHL are initially treated with rituximab in association with CHOP or CHOP-like regimens. In these patients, the role of rituximab in further salvage treatment remains to be determined. In the GEL/TAMO report by Martín and colleagues [40], no significant differences in response rates were documented in multivariate analysis between patients treated with R-ESHAP and previously exposed or not to rituximab. However, patients who had received prior rituximab had a significantly worse PFS and OS than rituximab naïve patients. Furthermore, prior treatment with this monoclonal antibody was also an independent adverse prognostic factor for both PFS and OS. In the experience of Fenske et al. [41], the administration of rituximab given with first-line or salvage therapy prior to HDT/ASCT was associated with PFS and OS at 3 years superior to that observed when this monoclonal antibody was not employed during the entire therapeutic patient history. In the CORAL trial [42], the response rates after salvage therapy were affected by several independent factors. These include saaIPI score, short relapse time from diagnosis (<12 months), and prior rituximab treatment. These same independent factors negatively influenced the 3-year EFS, PFS, and OS. However, patients relapsing after more than 12 months from diagnosis benefited from the introduction of rituximab into their salvage regimen and showed 3-year EFS ranging from 40% to 50%. In conclusion, at present, the optimal second-line regimen is not defined, and the benefit of the inclusion of standard dose of rituximab in salvage therapy for patients previously exposed to this agent is also unclear although known risk factors might be useful in choosing salvage therapeutic strategy. These factors include saaIPI, response (CR versus PR refractory) to upfront therapy disease status (early versus late relapse) at the time of salvage therapy. The third aspect regards the development of resistance to rituximab. One possibility in overcoming this resistance consists in using high-dose (HD) of this antibody. This therapeutic aspect was evaluated by Khouri et al. [43]. HD-rituximab (HD-R) was employed after mobilization chemotherapy and again on day 1 and day 8 after HDT/ASCT. In this study, the HDT consisted of standard BEAM. Fifty-nine patients (88%) were exposed before to rituximab during salvage chemotherapy. The median time from last rituximab dose to study enrollment was 38 days. The results of this experience indicate that HD-R combined with HDT/ASCT is feasible and effective treatment in relapsed patients previously treated with immunotherapy. 

 An attempt to develop more effective therapeutic strategy for relapsed DLBCL patients consists in the combination of radioimmunotherapy (RIT) with the standard chemotherapy conditioning regimens. After Press et al. [44], first established the feasibility of high-dose RIT with ASCT, several studies have used myeloablative RIT with promising results. The RIT combined with high-dose chemotherapy was superior compared to historical data especially in the salvage of patients with high IPI scores and residual PET-avid disease [45]. To further increase the therapeutic potential of RIT, Winter et al. [46] tested dose-escalated ^90^Y-ibriyumumab tiuxetan combined with BEAM and ASCT. In this study, 30% and 36% of the 44 treated patients had achieved less than a PR to their most recent treatment or never had obtained CR. Thus, respectively, 30% of cases would not have been eligible for HDT/ASCT at most centers. The estimated 3-years PFS and OS reported in this unfavorable series of patients were 43% and 60%, respectively. Careful dosimetry rather than weight-based strategy for dose escalation was required to avoid toxicity and under treatment.

Finally, one relevant prognostic factor associated with DLBCL consists of the cell origin of malignant cells [47–51]. In fact, the gene expression profile (GEP) resembling that of germinal center B cells (GCB) is predictive of better patient outcome than a profile resembling that of activated B cells (ABC). Cell-of-origin (COO) algorithms [52, 53] can also translate GEP data into practical applications. In the prerituximab era, studies using conventional dose therapy or HDT/ASCT concluded in favour of predictive prognostic value of COO [48, 51]. In contrast, the clinical significance of DLBCL subtyping, as defined by COO, is more controversial in patients treated at diagnosis with immunochemotherapy [53–56]. At relapse, few data regarding the clinical impact of COO-subsets are available. Recently a subanalysis of Coral trial [57] has indicated that COO retains its prognostic value in relapse/refractory DLBCL patients. In addition, a better response to R-DHAP was documented in GCB-like DLBCL cases. In contrast with these findings, in the study by Gu et al. [58], COO failed to predict survival in DLBCL patients, either with chemosensitive or chemoresistant disease, treated with HDT/ASCT. Further studies are needed to clarify the predictive value of DLBCL subtyping in the setting of patients with refractory/relapsing disease.

In conclusion, the benefit of HDT/ASCT for refractory or relapsed DLBCL is restricted to patients with immunochemosensitive disease. In fact, the response to second-line treatment seems to predict patient outcome after HDT/ASCT.

 Different therapeutic approaches are required to salvage patients with disease resistant to rituximab and chemotherapy. New agents such anti-CD20 antibodies therapeutically more active than rituximab, radiolabeled-antibodies, histone deacetylase inhibitors, various molecules which target mTOR, inhibitor of protein Kinase C*β*, and other types of target therapy might be effective in controlling refractory-relapsing DLBLC.

## Figures and Tables

**Table 1:** Phase III trials of HDT/ASCT in CR or PR unfavorable NHL patients.

Author	Year	*n*	Histological classification	DLCL (%)	Immunological phenotype (%)	aaIPI ≥ 2 (%)	Disease status HDT/ASCT	Therapy	Shorten induction Yes/No	PFS/EFS (%)	*P*	OS (%)	*P*
Verdonk [12]	1995	35 34	W.F.	26 33	B: 77 B: 79	44 44	PR	CHOP × 8 versus CHOP × 4 + HD-CTX-TBI/ASCT	Yes	4y: 53 4y: 41	N.S.	4y: 85 4y: 56	N.S.

Martelli [13]	1996	27 22	W.F./Kiel	62 40	B: 70 B: 45	N.R.	PR	DHAP^1^ × 6 versus BEAC^1^/ASCT	No	5y: 52 5y: 73	N.S.	59 73	N.S.

Haioun [14]	2000	111 125	W.F.	61 56	B: 63 B: 60	90 80	CR	ACVB versus ACVB + CBV/ASCT	No	8y: 39 8y: 55	0.02	8y: 49 8y: 64	0.04

Kluin-Nelemans [15]	2001	56 49	REAL	58 50	B: 55 B: 66	29 31	CR, PR	ChVmP/BV^1^ × 8 versus ChVmP/BV^1^ × 6 + BEAM/ASCT	Yes	5y: 56 5y: 61	N.S.	5y: 77 5y: 68	N.S.

Milpied [16]	2004	99 98	W.F.	74 77	B: 74 B: 77	49 57	PR	ACBVP^2^ versus CEOP + ECVBP^2^ + BEAM/ASCT	No	5y: 37 5y: 55	0.037	5y: 56 5y: 71	N.S.

^1^Plus radiotherapy at bulky disease.

^2^Plus radiotherapy at bulky disease and intrathecal prophylaxis in very high-risk patients.

W.F.: working formulation—NHL classification; Kiel: Kiel classification of NHL; CR: complete response; PR: partial response; CHOP: cyclophosphamide, doxorubicin, vincristine, and prednisone; HD-CTX: high-dose cyclophosphamide; TBI: total body irradiation; DHAP: cisplatin, cytarabine, and high-dose dexamethasone; BEAC: carmustine, etoposide, cytarabine, and cyclophosphamide; ACBV: doxorubicin, cyclophosphamide, vindesine, and bleomycin; CBV: cyclophosphamide, vinblastine, and bleomycin; ChVmP/BV: cyclophosphamide, doxorubicin, teniposide, prednisone, bleomycin, and vincristine; BEAM: carmustine, etoposide, cytarabine, and melphalan; CEOP: cyclophosphamide, epirubicin, vincristine, and prednisone; ECVBP: epirubicin, cyclophosphamide, vindesine, bleomycin, and prednisone; N.S.: not significant.

**Table 2:** Phase III trials of HDT/ASCT in unfavorable NHL patients.

Author	Year	*n*	Histological classification	DLCL (%)	Immunological phenotype (%)	aaIPI ≥ 2 (%)	Disease status HDT/ASCT	Therapy	Shorten induction yes/no	PFS/EFS (%)	*P*	OS (%)	*P*
Gianni [17]	1997	58 40	W.F.	88 91	N.R.	74 94	CR, CRu, PR, SD, MR, PD	MACOP-B^2^ versus HDS^#^ + mito-L-PAM^2^/ASCT	No	7y: 49 7y : 76	<0.001	7y: 55 7y : 81	0.09

Santini [18]	1998	61 63	W.F.	72 77	B: 75 B: 83	59 54	CR, CRu, PR, SD, MR, PD	VACOP-B^1^ versus VACOP-B^1^ + BEAM/ASCT	No	6y: 48 6y: 60	N.S.	6y: 65 6y : 65	N.S.

Gisselbrecht [19]	2002	181 189	Kiel/WHO 1999	62.5 60	B: 79 B: 75	97 99	CR, CRu, PR, SD, MR, PD	ACBVP^3^ versus CEOP^3^ + ECVBP + BEAM/ASCT	Yes	5y: 52 5y: 39	0.01	5y: 60 5y : 46	0.007

Kaiser [20]	2002	154 158	REAL	61 58	B: 79 B: 73	75 73	CR, CRu, PR, SD, MR, PD	CHOEP^1^ × 5 versus CHOEP^1^ × 3 + BEAM/ASCT	Yes	3y: 49 3y: 59	N.S.	3y: 63 3y: 62	N.S.

Martelli [21]	2003	75 75	REAL	84 78	B: 81 B: 70	100 100	CR, CRu, PR, SD, MR, PD	MACOP-B versus MACOP-B + BEAC/ASCT	Yes	5y: 49 5y: 61	N.S.	5y: 65 5y: 64	N.S.

Olivieri [22]	2005	106 116	W.F.	78 75	B: 83 B: 80	68 72	CR, CRu, PR, SD, MR, PD	VACOP-B^1^ × 12 weeks versus VACOP-B^1^ × 8 weeks + HD-CTX + HD-VP16 + BEAM/ASCT	No	7y: 44.9 7y: 40.9	N.S.	7y: 60 7y : 57	N.S.

Vitolo [23]	2005	66 60	REAL	90 80	B: 96 B : 90	80 87	CR, CRu, PR, SD, MR, PD	Mega CEOP^2^ × 6–8 versus HDS^#^ + mito-L-PAM^2^/ASCT	No	6y: 48 6y: 45	N.S.	6y: 63 6y: 49	N.S.

Betticher [24]	2006	59 70	REAL	69 76	B: 74 B: 93	88 72	CR, CRu, PR, SD, MR, PD	CHOP^2^ × 8 versus HDS^#^ + mito-L-PAM^2^/ASCT	No	3y: 33 3y: 39	N.S.	3y: 53 3y: 46	N.S.

Lynch [25]	2010	234 233	W.F.	N.R.	N.R.	98 98	CR, CRu, PR, SD, MR, PD	CHOP^1^ × 6–8 versus CHOP^1^ × 3 + BEAM/ASCT	Yes	5y: 38 5y: 44	N.S.	5y: 50 5y: 50	N.S.

^1^Plus radiotherapy at bulky disease.

^2^Plus radiotherapy at bulky disease and intrathecal prophylaxis in very high-risk patients.

^3^Plus intrathecal prophylaxis in very high-risk patients.

^#^See [17, 23, 24].

W.F.: working formulation-NHL classification; Kiel: Kiel classification of NHL; WHO: World Health Organization classification of NHL; CR: complete response; CRu: unconfirmed complete response; PR: partial response; MR: minor response, SD: stable disease, PD: progressive disease; MACOP-B: methotrexate with leucovorin rescue, doxorubicin, cyclophosphamide, vincristine, prednisone, and bleomycin; HDS: high-dose sequential chemotherapy; mito-L-PAM: mitoxantrone and melphalan; VACOP-B: etoposide, doxorubicin, cyclophosphamide, vincristine, prednisone, and bleomycin; BEAM: carmustine, etoposide, cytarabine, and melphalan; ACBVP: doxorubicin, cyclophosphamide, vindesine, bleomycin, and prednisone; (mega) CEOP: cyclophosphamide, epirubicin, vincristine, and prednisone; ECVBP: epirubicin, cyclophosphamide, vindesine, bleomycin, and prednisone; CHOEP: cyclophosphamide, doxorubicin, vincristine, etoposide, and prednisone; BEAC: carmustine, etoposide, cytarabine, and cyclophosphamide; HD-CTX: high-dose cyclophosphamide; HD-VP16: high-dose etoposide; N.R.: not reported; N.S.: not significant.

**Table 3:** Studies of HDT/ASCT in unfavorable DLBCL patients.

Author	Year	*n*	Pathological phenotype	DLCL (%)	Immunological phenotype (%)	aaIPI ≥ 2 (%)	Therapy	Shorten induction yes/no	PFS/EFS (%)	OS (%)
Tarella [27]	2007	112	REAL	79	B. 100	100	Modified R-HDS^ # 1^	No	4y: 73	4y: 76

Vitolo [28]	2009	97	REAL	86	B. 100	100	R-mega CEOP14 × 4 + R-MAD^2^ × 2 + BEAM/ASCT	No	4y: 73	4y: 80

Dilhuydy [29]	2010	42	REAL	N.R.	B. 100	100	R × 4 + CEEP × 2 + R-MTX/R-MC + BEAM/ASCT	Yes	5y: 55	5y: 74

Fitoussi [30]	2011	209	WHO	N.R.	B. 100	100	R-ACVBP × 4 + BEAM/ASCT	Yes	4y: 76	4y: 78

^1^Plus radiotherapy at bulky disease.

^2^Plus radiotherapy at bulky disease and intrathecal prophylaxis in very high-risk patients.

^#^See [26].

REAL: revised European-American lymphoma classification; WHO: World Health Organization classification of NHL; R: rituximab; (mega) CEOP: cyclophosphamide, epirubicin, vincristine, and prednisone; MAD: mitoxantrone, cytarabine, and dexamethasone; BEAM: carmustine, etoposide, cytarabine, and melphalan; CEEP: cyclophosphamide, epirubicin, vindesine, and prednisone; MTX: methotrexate; MC: methotrexate and cytarabine; ACBVP: doxorubicin, cyclophosphamide, vindesine, bleomycin, and prednisone; N.R.: not reported.

**Table 4:** Rituximab-based salvage therapy in rituximab-naïve relapsing/refractory DLBCLs.

Author	Year	*n*	Pathological phenotype	DLCL (%)	Therapy	Conditioning regimen	PFS/EFS (%)	*P*	OS (%)	*P*
Kewalramani [36]	2004	36 147	WHO	100	R-ICE ICE	*	2y: 54 2y: 43	N.S.	2y: 67 2y: 56	N.S.

Sienawski [37]	2007	19 19	WHO	80	R-DHAP^1^ DHAP^1^	BEAM	2y: 57 2y: 18	0.0051	2y: 77 2y: 37	0.0051

Vallenga [38]	2008	113 112	WHO	80.5 78.6	R-DHAP-VIM-DHAP^ 1^ DHAP-VIM-DHAP^ 1^	BEAM	2y: 52 2y: 31	0.002	2y: 59 2y: 52	N.S.

Mounier [39]	2011	470	WHO	100	N.R.	BEAM and others^#^	5y: 48	0.001**	5y: 63	N.R.

^1^Plus radiotherapy at bulky disease.

*The choice of conditioning regimen depended on the patient's age, the extent of previous therapy and the clinical trials active at the time of transplantation (see [30]).

^#^See [39].

**Each patient was assessed as his or her own control.

WHO: World Health Organization classification of NHL; R: rituximab; ICE: ifosfamide, carboplatin, and etoposide; DHAP: cisplatin, cytarabine, and dexamethasone; VIM: etoposide, ifosfamide, and methotrexate; BEAM: carmustine, etoposide, cytarabine, and melphalan; N.R.: not reported; N.S.: not significant.

**Table 5:** Salvage therapy in relapsing/refractory DLBCLs previously exposed to rituximab.

Author	Year	Kind of study	*n*	Pathological phenotype	DLCL (%)	Therapy	Conditioning regimen	PFS/EFS (%)	*P*	OS (%)	*P*
Martín [40]	2008	Retrospective	94 69	WHO	100	R-ESHAP (prior R) R-ESHAP (no prior R)	*	3y: 17 3y: 57	0.008	3y: 38 3y: 67	0.004

Fenske [41]	2009	Retrospective	818 176	WHO	100	R-CT (no prior R) R-CT (prior R)	*	3y: 50 3y: 38	0.008	3y: 57 3y: 45	0.006

Gisselbrecht [42]	2010	Perspective	194 202	WHO	100	R-DHAP R-ICE	BEAM	3y: 42 3y: 31	N.S.	2y: 51 2y: 47	N.S.

*The choice of conditioning regimen depended on the patient's age, the extent of previous therapy, and the clinical trials active at the time of transplantation (see [40, 41]).

WHO: World Health Organization classification of NHL; R: rituximab; ESHAP: etoposide, methylprednisolone, cisplatin, and cytarabine; CT: multiple variable regimes; ICE: ifosfamide, carboplatin, and etoposide; DHAP: cisplatin, cytarabine, and dexamethasone; BEAM: carmustine, etoposide, cytarabine, and melphalan; N.S.: not significant.
